# Developing a Novel Immune-Related Seven-Gene Signature and Immune Infiltration Pattern in Patients with COVID-19 and Cardiovascular Disease

**DOI:** 10.3390/jcdd9120450

**Published:** 2022-12-09

**Authors:** Yajuan Fu, Juan Zhang, Lingbo Xu, Hui Zhang, Shengchao Ma, Yujing Gao, Yideng Jiang

**Affiliations:** 1 National Health Commission Key Laboratory of Metabolic Cardiovascular Diseases Research, Ningxia Medical University, Yinchuan 750004, China; 2 Ningxia Key Laboratory of Vascular Injury and Repair Research, Ningxia Medical University, Yinchuan 750004, China; 3 Department of Pathophysiology, School of Basic Medical Sciences, Ningxia Medical University, Yinchuan 750004, China

**Keywords:** cardiovascular disease, COVID-19, immune signature, hub immune-related genes, small molecular agents

## Abstract

Background: patients with pre-existence of cardiovascular disease (CVD) are vulnerable to coronavirus disease 2019 (COVID-19), and COVID-19 will cause long-term burden of CVD. However, the common pathogenic mechanisms are not fully elucidated. More detailed knowledge of linking biological molecules and the role of immune signature would allow more valuable and specific clinical management. Methods: the gene expression profiles of CVD and COVID-19 were retrieved from the GEO database. Common differentially expressed genes (DEGs) were screened with the Limma R package and the WGCNA algorithm, and then functional enrichment analysis, protein-protein interaction network, hub genes, and small therapeutic molecules analyses were performed. The hub immune-related genes (HIRGs) were intersected, and their associations with immune cells, expressional correlation, evaluated performance, and potential signal pathways were further investigated. Results: In total, 57 common DEGs were identified as a shared transcriptional signature between CVD and COVID-19, and 12 hub genes were screened using five topological algorithms. There are common altered immune responses in the response of these two diseases, and seven HIRGs, including C5AR1, MMP9, CYBB, FPR2, CSF1R, TLR2, and TLR4, were identified, with positive correlation to altered macrophages and neutrophils. Nine small molecular agents (SMAs) were detected as promising therapeutic drugs. These seven HIRGs mainly participated in the inflammatory immune response through activation of Il2 stat5 signaling and Tnfa signaling via nfκb pathways, and ROC curves confirmed their good discriminatory capacity in the two diseases. Conclusions: this study established the co-expression network and identified a new immune-related seven-gene signature as therapeutic targets, which may provide new insights into pathogenic mechanisms and novel clinical management strategies.

## 1. Introduction

Cardiovascular disease (CVD), especially ischaemic heart disease (IHD), which is also referred to as coronary artery disease (CAD), is the leading cause of global death (responsible for 16% of the world’s total mortality) and disability and has been the largest increase in deaths since 2000, with deaths rising by more than 2 million and up to 890 in 2019 [1,2]. Many risk factors, such as lifestyle-associated factors (smoking, obesity, and alcohol consumption), air pollution, and family history contribute to CVD. Currently, several approaches for early detection of this disease in clinical practice, such as troponins and cardiac natriuretic peptides, have been developed [3]. However, approaches with adequate accuracy or specificity are still lacking, and increasing concern for a broader range of therapeutic agents and reducing the residual risks is of great interest.

The coronavirus disease 2019 (COVID-19) has been spreading astonishingly and caused catastrophic losses worldwide. Prevalence and genetic variation are continuing to threaten humans’ lives and to provoke severe anxiety [4]. COVID-19 patients with comorbidities, especially with preexisting CVD, are at a high risk level of a more severe course and even death [5]. Most recently, several studies have demonstrated that arterial and venous thromboembolic events (VTE) immediately increased after severe COVID-19, and thromboembolic disease increased mortality during the COVID-19 pandemic [6,7,8,9,10]. Besides, the ACE2 receptor, responsible for the severe acute respiratory syndrome coronavirus 2 (SARS-CoV-2) entry, also was reported as being expressed on the arterial and venous endothelium [11], which may partly explain higher incidences of heart failure-related events and acute coronary syndromes among individuals with COVID-19. Studies also have found that dysregulated systemic inflammation, which can be stimulated through many approaches, such as TLRs (TLR2 and TLR4) -mediated signal pathways or C5a-C5aR1 axis, could weaken myocardial function and harm the organs [12,13]. Other hypotheses, such as arterial hypoxia and coagulopathy, are also proposed to explain the interplay between CVD and COVID-19. Nonetheless, the underlying mechanism linking CVD with COVID-19 is still not fully elucidated, and novel simple strategies to reduce CVD events in patients with COVID-19 are needed. Effective tools-RNA-seq and microarray technologies—have been implemented to investigate pathogenesis and explore therapeutic targets for the disease. In this study, we aimed to investigate the pathogenetic and genetic correlation between CVD and COVID-19. The datasets GSE6630 and GSE164805 were retrieved from GEO database, and common shared DEGs were screened based on the Limma R package and WGCNA algorithms. Then, the hub genes were identified by the protein–protein interaction network, and immune-related hub genes (IRHGs) were further intersected. Finally, the relationship between IRHGs and immune cells was measured with ssGSEA algorithms, and their diagnostic sensitivity was evaluated. In conclusion, our study illustrated the potential molecular biological mechanisms underlying different disorders and revealed the immune infiltration patterns in critical illnesses, which we hope will provide new insights into the treatment of COVID-19 patients with CVD.

## 2. Materials and Methods

### 2.1. Microarray Datasets Collection and Preprocessing

Microarray profiles associated with CVD and COVID-19 were obtained from the GEO database (https://www.ncbi.nlm.nih.gov/geo/ (accessed on 22 July 2022), which contains high-throughput gene expression profiles submitted by research institutions. The GSE66360 series (GPL570 platform), contains gene expression data of circulating endothelial cells from 49 patients experiencing acute myocardial infarction and 50 healthy cohorts [14], and this also includes the GSE164805 dataset (GPL26963 platform), which includes transcriptome data of peripheral blood mononuclear cells from COVID-19 patients (n = 10) and healthy controls (n = 5) [15]. The robust multiarray average (RMA) method [16] was used to remove batch effects, and the Limma R package was used for normalization between groups of the series’ matrix files. Then, log2 transformation was performed, and probes and gene symbols were matched (the maximum was reserved in the presence of duplicate expression data), based on the corresponding annotation documents of platforms [17]. We used the Limma R package to distinguish differentially expressed genes (DEGs), and gens with Padj < 0.05 and |log2FoldChange (FC)| > 1 were screened as significant DEG. Volcano plots were constructed by the ggplot2 R package [18], and Venn diagrams were plotted with the online website (https://www.bioinformatics.com.cn/ (accessed on 22 July 2022).

Construction of co-expression networks was conducted with weighted gene co-expression network analysis (WGCNA).

WGCNA was performed with the WGCNA R package to construct the network, detect the module, and select highly correlated genes based on the coherence of gene sets and the correlation between gene sets and traits [19]. Firstly, we constructed the network by calculating the adjacency matrix, and the “pickSoftThreshold” function was employed to obtain the optimal soft power for matrix construction according to the scale-free topology criterion. Then, module detection was conducted using unsupervised clustering with densely interconnected genes as clusters, and the correlation between modules and genes was calculated. Modules with high trait significance were considered to relate to the sample trait, and genes in this high module membership were selected for further validation. In our study, the soft threshold β was 16 in the GSE 164,805 and 8 in the GSE66360 datasets, and the networkType = “signed”.

### 2.2. KEGG and GO Enrichment Analyses

To investigate the biological functional categories and the underlying mechanisms of the common gene set (CGS), we performed gene ontology (GO), and the Kyoto Encyclopedia of Genes and Genomes (KEGG) analyses using the web server (http://www.bioinformatics.com.cn/basic_local_go_pathway_enrichment_analysis_122 (accessed on 23 July 2022) was used. *p*-value < 0.05 was considered significant.

### 2.3. Establishment of Protein–Protein Interaction (PPI) Networks

PPI networks can predict associations between proteins not only at the physical interactions level, but also in the functional association aspects [20]. In this study, the online STRING database (http://string-db.org (accessed on 23 July 2022) was employed to construct PPI networks with a score (median confidence) >0.4 [21]. Then, we downloaded the interaction information and visualized it with Cytoscape software v3.8.1 [22], and the molecular complex detection (MCODE) plugin was applied to identify the key modules. Hub genes (top 15) using the five topological analysis methods, including degree, edge percolated component (EPC), maximumc neighborhood component (MNC), density of maximum neighborhood component (DMNC), and closeness, were screened by the Cytohubba plugin in Cytoscape [23].

### 2.4. Candidate Small Molecular Agents (SMAs) Prediction

To identify the potential SMA targeting in COVID-19 patients with CVD, the 12 hub genes were uploaded into the Broad Institutes Connectivity Map (cMAP) database (https://portals.broadinstitute.org/cmap (accessed on 23 July 2022) [24]. Top10 drug candidates are sorted by negative values.

### 2.5. Immune Cell Infiltration Evaluation and Its Correlation with Hub IRGs (HIRGs)

The immune checkpoint genes (ICGs) were collected from the literature [25], and immune enrichment scores of 28 immune cells were evaluated via the R package “GSVA” [26] through the single-sample gene set enrichment analysis (ssGSEA) method. By using the “corrplot” package, the expression matrix of Pearson correlation coefficients between each immune cell was visualized. Additionally, the correlation between seven HIRG expressions and immune cell infiltration was calculated with the “ggstatsplot” package, and then the “ggplot2” package was used for visualization.

### 2.6. Diagnostic Efficacy Evaluation of HIRGs and Their Expressional Correlation

Receiver operating characteristic (ROC) curve analysis was performed to evaluate the predictive efficiency on each HIRG, and the area under the curve (AUC) values were calculated using an online website (https://www.xiantao.love/products/ (accessed on 24 July 2022). HIRGs with AUC >0.7 were deemed as high-efficiency genes for disease diagnosis. “Corrplot”, a visualization package of a correlation matrix, was used to analyze the correlation of genes [27].

### 2.7. Gene Set Enrichment Analysis (GSEA)-Based Pathway Confirmation Study

To verify the common signaling pathway of the seven HIRGs in CVD and COVID-19, we performed GSEA analysis based on the expression levels of each gens using the R package clusterProfiler. The gene set h.all.v7.4.entrez.gmt was obtained from MSigDB (http://www.gsea-msigdb.org/gsea/msigdb/index.jsp (accessed on 25 July 2022) [28]. The gene sets were determined according to the enrichment score, and Padj <0.05 was set as a significance criterion.

## 3. Results

### 3.1. Co-Expression Genes Shared in CVD and COVID-19

**Figure 1:**
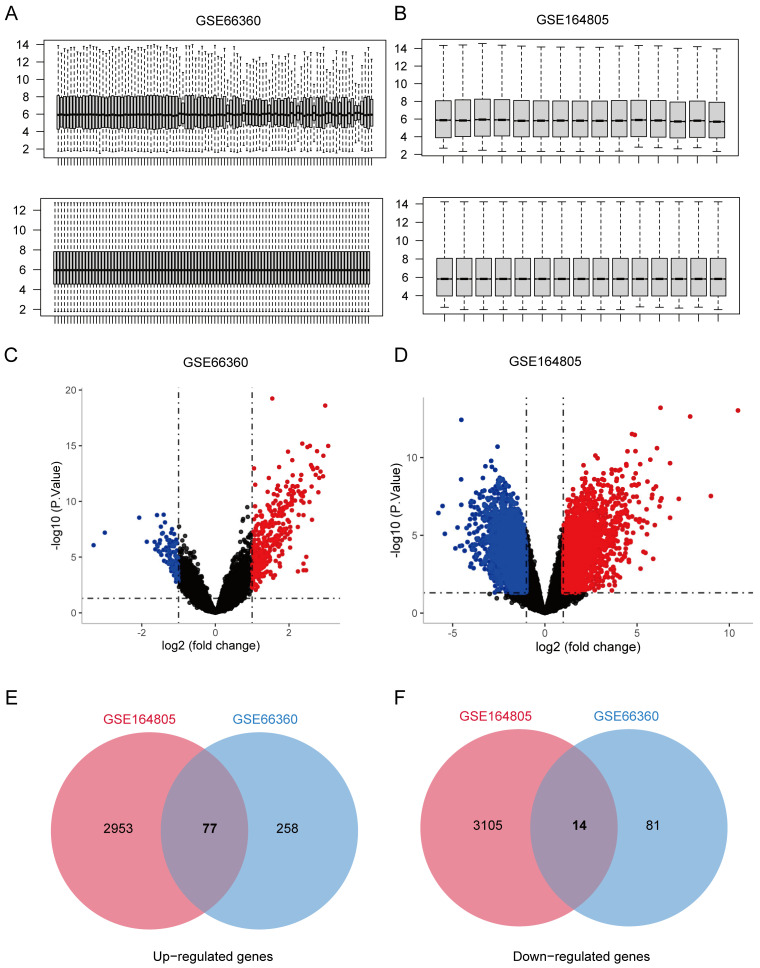
The shared differentially expressed genes between CVD and COVID-19. The boxplots for gene expression values before or after normalization using Limma R packages in (**A**) CVD-related GSE66360 dataset and (**B**) COVID-19-related GSE164805 dataset. Volcano plots of DEGs in (**C**) GSE66360 and (**D**) GSE164805 datasets, where genes with Padj < 0.05 and |logFC (fold change)| > 1 were considered as significant DEGs. The red dots present up-regulated genes, the green dots indicate down-regulated genes, and the black dots present non-significant genes. Venn diagrams of (**E**) up-regulated and (**F**) down-regulated DEGs in the two datasets are presented. The two datasets show an intersection of 91 DEGs.

To explore the co-expressed genes shared in CVD and COVID-19, microarray profiles were retrieved from the GEO database. We used R software (R v4.0.2) to normalize and logarithmatize the data, and then we deleted the probes with no annotation information (Figure 1A,B). According to the criteria (Padj <0.05 and |logFC| > 1), a total of 430 DEGs were identified from the GSE66360 dataset, including 335 up-regulated and 95 down-regulated genes (Figure 1C). In the GSE164805 dataset, 6149 DEGs (3030 up-regulated and 3119 down-regulated genes) were screened (Figure 1D). The crosstalk genes between the two datasets were intersected by Venn diagrams, and 77 up-regulated and 14 down-regulated genes were screened (Figure 1E,F), suggesting there might be similar pathogenesis in CVD and COVID-19.

### 3.2. WGCNA Reveals Co-Expression Modules Associated with CVD and COVID-19

**Figure 2:**
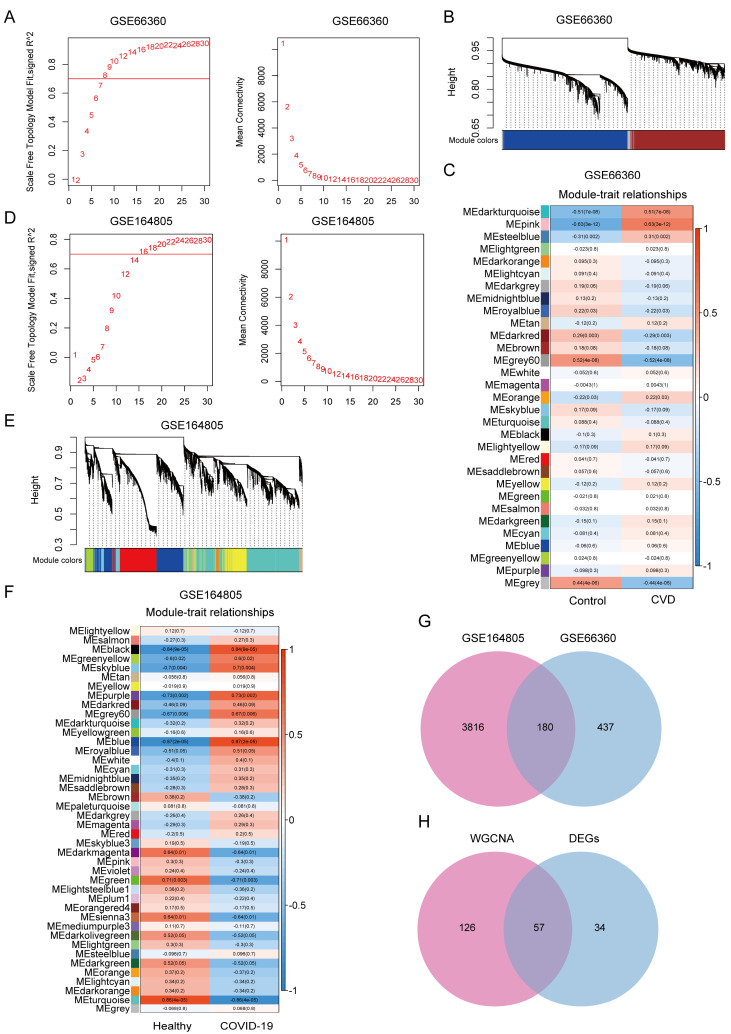
Construction of co-expressed gene networks in dataset GSE66360 and GSE164805. WGCNA analysis of key modules in (**A**–**C**) GSE66360 and (**D**–**F**) GSE164805. (**A**,**D**) Selection of soft threshold powers. (**B**,**E**) Cluster dendrogram of genes in CVD and COVID-19, where each color represents one gene module. (**C**,**F**) Heatmap depicting correlations between module eigengenes and sample traits. Each block contains the correlation value and *p*-value. The red indicates positive correction, and the blue presents negative correction between genes with traits. (**G**) Venn diagrams of shared genes between the modules of GSE66360 and GSE164805 datasets. (**H**) Venn diagrams showing 57 overlapped genes based on module genes and DEGs.

Using WGCNA, we further explored the co-expressed gene modules and gene networks involved in the development of CVD and COVID-19. For GSE66360, soft threshold β 8 was selected according to the results of a scale-free topology model and the mean connectivity (Figure 2A). The heat map showed the relationship between gene modules and clinical traits by using a hierarchical clustering algorithm and the Spearman correlation coefficient, and a total of 31 modules that were highly related to CVD were identified. The pink module significantly exhibited the highest positive correlations (r = 0.63, *p* = 3 × 10^−12^) with CVD, including 671 genes (Figure 2B,C). Similarly, we set the soft-threshold power to 16, and the height was set to 0.25, to establish the network in the COVID-19-related dataset GSE164805 (Figure 2D). Among the 42 identified modules, the blue module (r = 0.87, *p* = 2 × 10^−5^), containing 3996 genes, was positively correlated with COVID-19 (Figure 2E,F). The 180 common genes of the modules detected from GSE66360 and GSE164805 were overlapped (Figure 2G). Taken together with the DEGs that were identified by R Limma package, a total of 57 overlapped genes were obtained and defined as a common gene set (CGS) (Figure 2H), which were considered extremely related to the pathogenesis of CVD and COVID-19.

### 3.3. Functional Enrichment of the CGS

**Figure 3:**
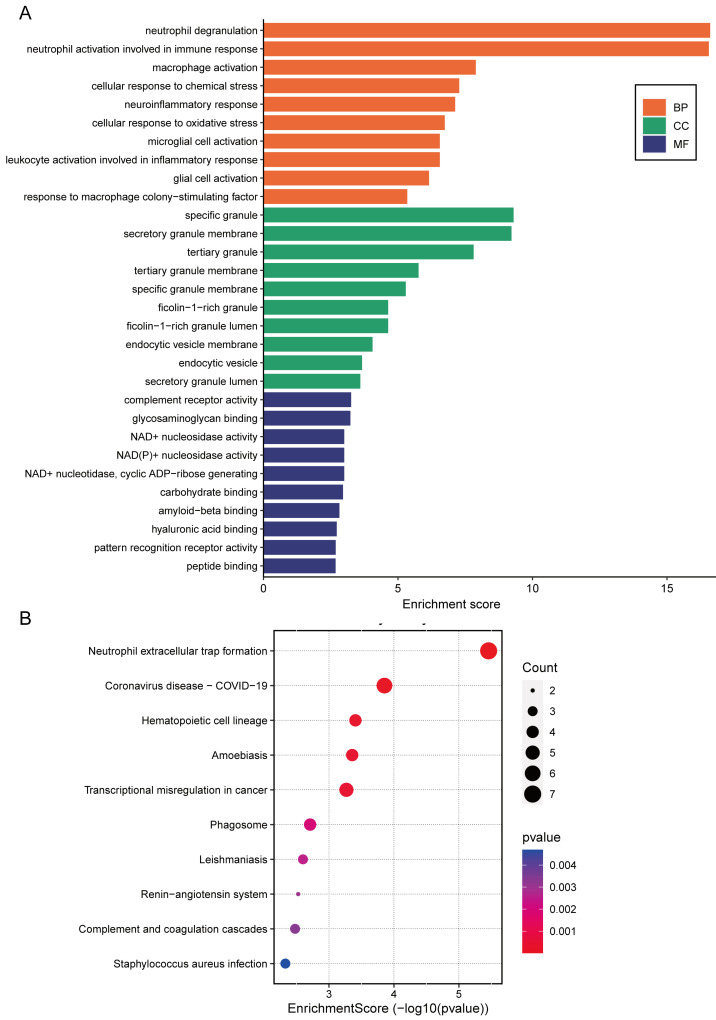
Gene ontology and pathway enrichment analysis. (**A**) GO and (**B**) KEGG enrichment analysis of the 57 common genes identified in CVD and COVID-19.

GO enrichment and KEGG pathway analyses were carried out to further understand the CGS functional categories. As shown in Figure 3A, in biological processes (BPs), the CGS was mostly enriched in neutrophil and macrophage activation, inflammatory immune response, and cellular response to oxidative stress. Among cellular components (CCs), most genes were mainly enriched in specific and tertiary granules, as well as the secretory and tertiary granule membranes; CGS was mostly involved in complement receptor activity, glycosaminoglycan binding, and NAD+ and NAD(P)+ nucleosidase activity of molecular functions (MFs). KEGG enrichment results demonstrated that neutrophil extracellular trap formation, coronavirus disease-COVID-19, and hematopoietic cell lineage pathways were significantly enriched (Figure 3B). These results forcefully indicated that inflammatory pathways and immune activation play vital roles in the progression of CVD and COVID-19.

### 3.4. Identification and Modular Analysis of Hub Genes

**Figure 4:**
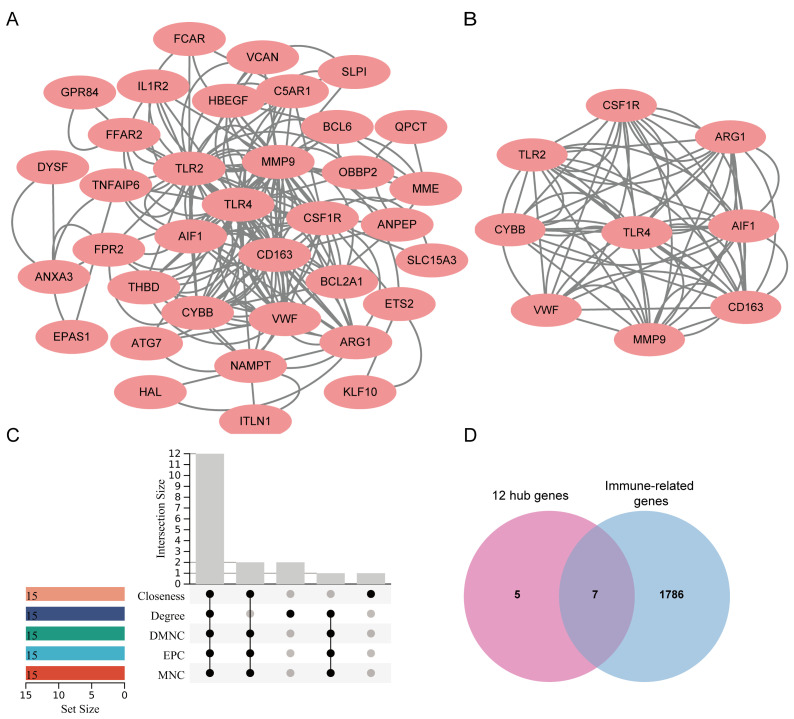
The PPI network and cluster analysis of common genes in CVD and COVID-19. (**A**) Cytoscape network visualization of 57 common genes based on the STRING online database. (**B**) The vital cluster was identified by the Cytoscape MCODE algorithm. (**C**) The 12 hub genes were identified according to five algorithms using cytoHubba. (**D**) Venn diagrams showing seven immune-related hub genes.

The complex PPI networks are helpful in disease gene identification, gene function prediction, and drug treatment identification [29,30]. Aiming at a target PPI will provide reliable and accurate information in cellular events, and it will also provide therapeutic potential. The CGS was uploaded into STRING for PPI network construction, followed by visualizing with Cytoscape software. As shown in Figure 4A, 36 nodes and 174 edges were displayed. The key cluster (9 nodes and 68 edges) was recognized with the MCODE algorithm based on the calculated scores (Figure 4B). Subsequently, the hub genes (top 15) were obtained using the CytoHubba plug-in based on five algorithms, including degree, EPC, MNC, DMNC, and closeness (Table 1), and 12 genes (TLR4, TLR2, MMP9, CD163, CSF1R, CYBB, VWF, AIF1, ARG1, THBD, C5AR1, and FPR2) were intersected as hub genes (Figure 4C). Thus, these 12 genes were considered as the kernel targets of CVD and COVID-19. Notably, the results of GO enrichment strongly suggested that the immune microenvironment was altered in the two diseases. Therefore, 1793 immune-related genes (IRGs) were retrieved from the Immunology Database and Analysis Portal database (ImmPort) (https://www.immport.org (accessed on 25 July 2022). After intersection with 12 hub genes, seven genes, including C5AR1, MMP9, CYBB, FPR2, CSF1R, TLR2, and TLR4, were identified as hub IRGs (HIRGs) (Figure 4D) (Table 2).

### 3.5. Identification of Candidate Drugs

Though a comprehensive method of antibiotics, oxygen therapy, and anticoagulants was applied to treat COVID-19 cases, it was not sufficient in many cases, and a large requirement is still needed for developing additional treatments. Therefore, we uploaded the 12 up-regulated hub genes to the cMAP database, and many potential therapeutic SMAs were identified. The SMAs with the highest absolute enrichment values were chosen (Table 3), including solanine, desoxypeganine, alpha-linolenic-acid, CAY-10577, homochlorcyclizine, altretamine, NU-1025, TG100-115, and raltegravir, suggesting their potential therapeutic effects on COVID-19 patients with CVD.

### 3.6. Immune Infiltrating Cell Analyses and Their Relationship with HIRGs

**Figure 5:**
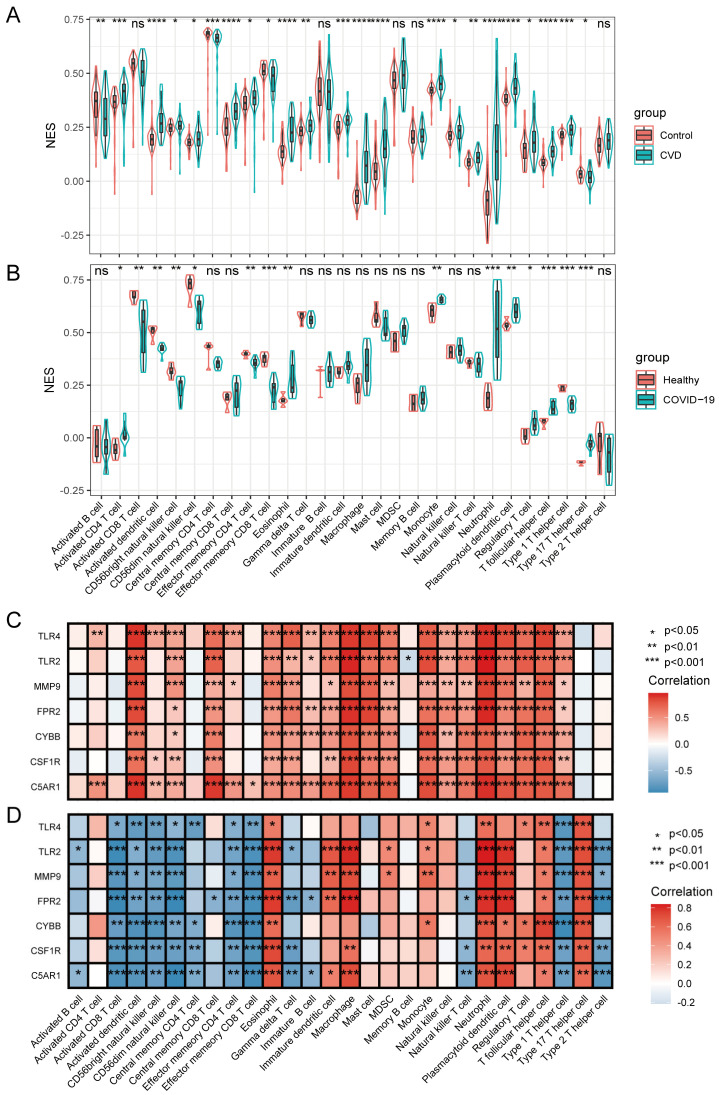
Immune-related hub gene signatures correlated with immune infiltrations. (**A**,**B**) Immune cell infiltration analysis in GSE66360 and GSE164805 datasets. (**C**,**D**) Correlation analysis between immune-related hub genes and infiltrating immune cells. * indicates *p* < 0.05, ** represents *p* < 0.01, *** represents *p* < 0.001, **** represents *p* < 0.0001, ns represents no significant difference.

Based on the results of the above-discussed functional enrichment analysis and verification of seven HIRGs, as well as the previous studies, immune response and inflammation were evidenced to play crucial roles in the progression of CVD and COVID-19. To investigate the differential immune landscape in the two diseases, ssGSEA was used to estimate the fraction of 28 immune cells among the healthy and patient groups. Specifically, the immune cell types in the CVD-related dataset were activated by CD4 T cells, activated dendritic cells, central memory CD8 T cells, eosinophils, immature dendritic cells, macrophages, mast cells, monocytes, neutrophils, plasmacytoid dendritic cells, T follicular helper cells, and type 1 T helper cells (Figure 5A). The immune cell types, including the eosinophil, monocyte, neutrophil, plasmacytoid dendritic cell, T follicular helper cell, and Type 17 T helper cell, were involved in the progression of COVID-19 (Figure 5B). These results exhibited a similar spectrum of immune cell fractions between CVD and COVID-19. We further evaluated the association between the seven HIRGs (C5AR1, MMP9, CYBB, FPR2, CSF1R, TLR2, and TLR4) and the immune cells. As shown in Figure 5C, the expression of HIRGs was mainly positive, with activated dendritic cells, macrophages, mast cells, monocytes, neutrophils, plasmacytoid dendritic cells and T follicular helper cells in the CVD dataset. TLR2, MMP9, FPR2, CSF1R, and C5AR1 expressions in COVID-19 were positively correlated with eosinophils, immature dendritic cells, macrophages, neutrophils, plasmacytoid dendritic cells, and Type 17 T helper cells (correlation > 0.75); TLR4 and CYBB expressions were positively correlated with Type 17 T helper cells and T follicular helper cells, suggesting their roles in regulating immunity (Figure 5D). Taken together, these results suggested that there are common altered immune responses implicated in CVD and COVID-19, and the identified seven HIRGs might contribute to the immune microenvironment of the two diseases.

### 3.7. Diagnostic Performance and Correlation Analysis of the HIRGs

**Figure 6:**
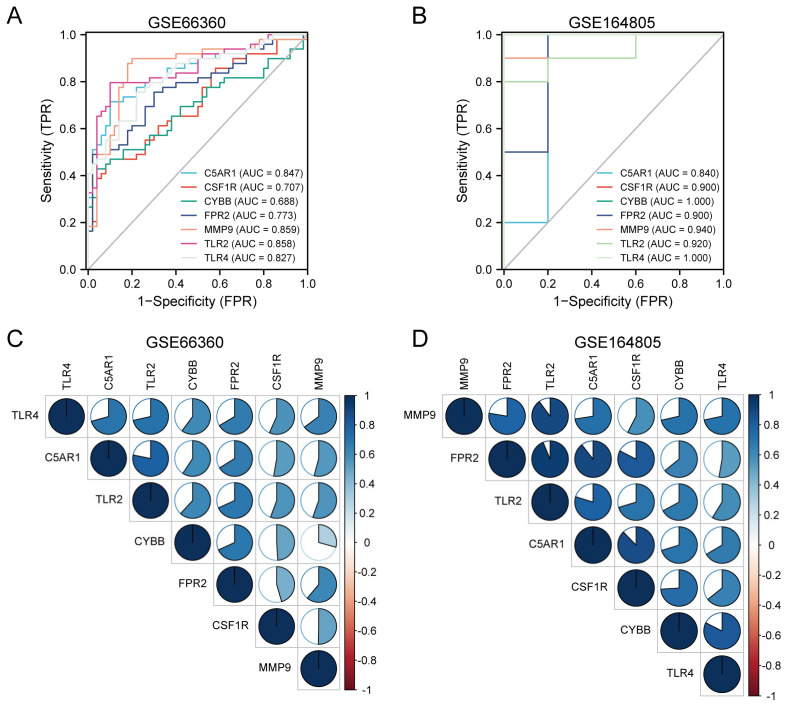
The diagnostic efficacy verification of seven immune-related hub genes and their expressional correlation. (**A**,**B**) ROC curves of seven immune-related hub genes in GSE66360 and GSE164805 datasets. (**C**,**D**) Reciprocal correlations between the seven immune-related hub genes. The area size and the color of circles represent the strength of the correlation between genes. The larger the size and the bluer the color, the stronger the correlation.

The diagnostic performance of the identified HIRGs was evaluated using ROC curves based on the expression data. In the CVD-related dataset GSE66360, the AUCs of C5AR1, MMP9, CYBB, FPR2, CSF1R, TLR2, and TLR4 were 0.847, 0.859, 0.688, 0.773, 0.707, 0.858, and 0.827, respectively (Figure 6A). Furthermore, the AUC values of these seven HIRGs were greater than 0.8 in the COVID-19-related dataset GSE164805 (Figure 6B). In addition, these genes exhibited significant reciprocal positive correlations, such as TLR2/C5AR1 (r = 0.78), C5AR1/TLR4 (r = 0.71) and TLR2/TLR4 (r = 0.71) in GSE66360 (Figure 6C), FPR2/MMP9 (r = 0.78), TLR2/MMP9 (r = 0.9), C5AR1/MMP9 (r = 0.73), TLR2/FPR2 (r = 0.94), C5AR1/FPR2 (r = 0.89), TLR2/C5AR1 (r = 0.8), CSF1R/C5AR1 (r = 0.88), CYBB/TLR4 (r = 0.82), and CSF1R/FPR2 (r = 0.83) in GSE164805 (Figure 6D). Given the above, these seven HIRGs hold a powerful discrimination capability and might be promising prevention and treatment targets for COVID-19 patients with CVD.

### 3.8. GSEA Identifies Seven HIRGs Associated Signaling Pathway

**Figure 7:**
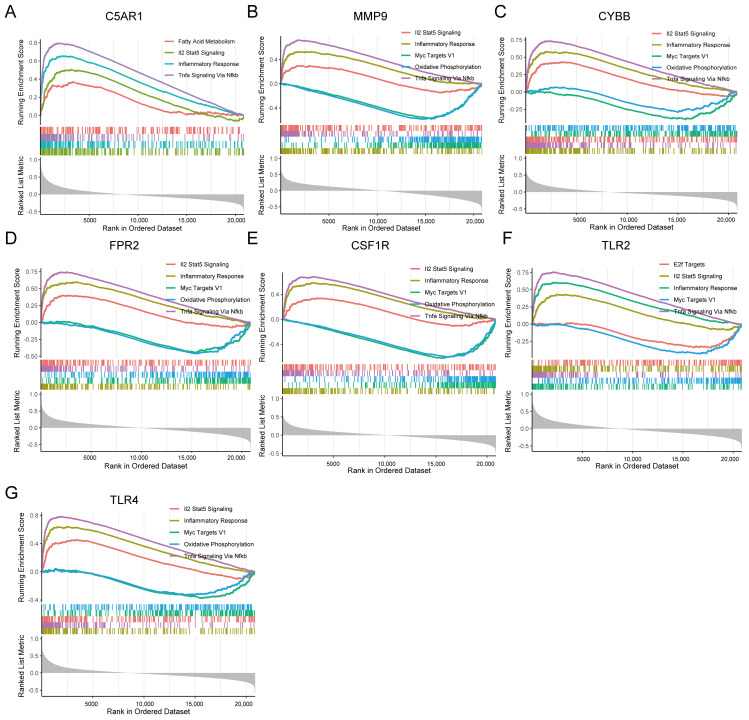
Gene set enrichment analysis reveals pathways of the seven-hub immune-related genes involved in CVD. (**A**) C5AR1; (**B**) MMP9; (**C**) CYBB; (**D**) FPR2; (**E**) CSF1R; (**F**) TLR2; (**G**) TLR4.

**Figure 8:**
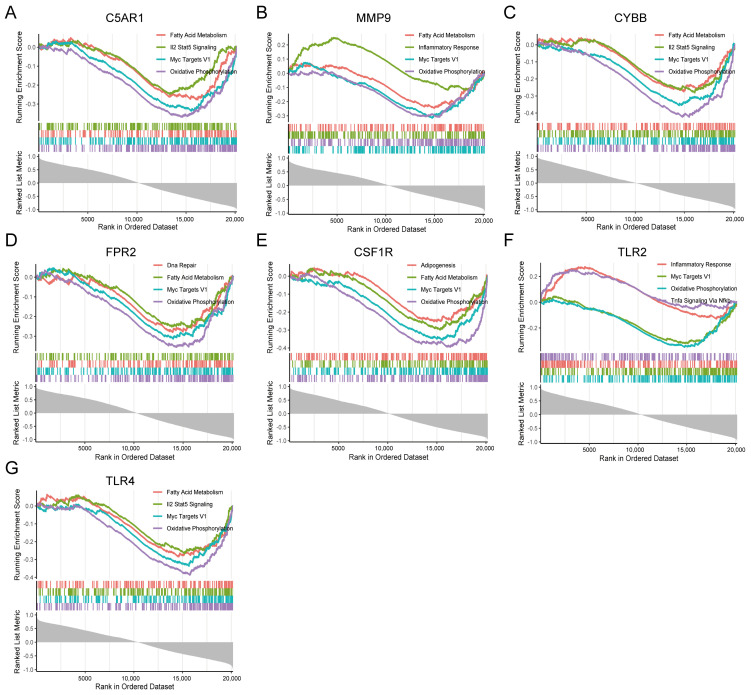
Gene set enrichment plot demonstrates the signaling pathways of the seven-hub immune-related genes involved in COVID-19. (**A**) C5AR1; (**B**) MMP9; (**C**) CYBB; (**D**) FPR2; (**E**) CSF1R; (**F**) TLR2; (**G**) TLR4.

To identify the signaling pathways of the seven immune-related genes in CVD and COVID-19, GSEA between high-and low-C5AR1, MMP9, CYBB, FPR2, CSF1R, TLR2, and TLR4 expression matrices was performed to recognize signaling pathways based on h.all.v7.4.entrez.gmt, collected in MSigDB. The results indicated that inflammatory response and Il2 stat5 signaling pathways were activated, while oxidative phosphorylation, fatty acid metabolism, and Myc targets V1 pathways were inhibited in the high-expression matrix in the COVID-19-related dataset (Figure 7). Meanwhile, GSEA results in GSE66360 exhibited activated KEGG items, including inflammatory response, Il2 stat5 signaling, and Tnfa signaling via nfκb pathways, as well as inhibited oxidative phosphorylation, and Myc targets V1 pathways (Figure 8). The results revealed that these pathways are positively or negatively associated with the seven immune-related genes, which were responsible for the regulation of immune inflammatory response in CVD and COVID-19. Taken together, these findings suggest that these signaling pathways, which are especially important in the development of the two diseases, may be the potential targets for the treatment of COVID-19 patients with CVD.

## 4. Discussion

COVID-19 with pre-existing CVD has a higher risk of mortality, and COVID-19 also can deteriorate the coexisting CVD or lead to complications in the cardiovascular system. Studies have found that COVID-19 profoundly affects CVD through viral toxicity, cardio-renal-pulmonary damage, endothelial cell damage, thromboinflammation, oxygen supply-demand mismatch, and cytokine storm [31], but the underlying mechanisms still have not been completely elucidated. In this study, we focused on the linking genetic signatures, the potential regulatory targets and pathways, and the possible therapeutic molecules to help to replenish the therapeutic management strategies.

In the present study, we screened 57 shared DEGs as CGSs between CVD and COVID-19, and functional enrichment analysis demonstrated that inflammatory pathways and immune activation were involved in the progression of the two diseases. Seven HIRGs, including C5AR1, MMP9, CYBB, FPR2, CSF1R, TLR2, and TLR4, were identified among the 12 hub genes identified based on the PPI networks. C5AR1 (also known as CD88), is a receptor of chemotactic and inflammatory peptide anaphylatoxin C5a. The C5a-C5aR1 axis participates in the pathophysiology of COVID-19, and blockade of the axis can limit the infiltration of myeloid cells and prevent excessive inflammation [12,32]. Besides, mitochondrial C5aR1 was evidenced to modulate the production of IL-1β and inflammatory gene signatures [33], and the C5a-C5aR1 axis controls tissue neovascularization [34]. The expression of MMP9 is increased in COVID-19 patients, and MMP9 plus other genes is associated with patients’ mortality [35]. CYBB, also named NADPH oxidase (mainly Nox2), was proven to play a crucial role in vascular and cerebral oxidative stress and inflammation [36], and Nox2 activation stimulates oxidative stress and associates with a severe course and thrombotic events in COVID-19 patients [37]. FPR2 (also named lipoxin A4 receptor (LXA4R, ALX) is involved in the development of chronic inflammatory diseases, such as atherosclerosis, Alzheimer’s disease, colitis, and NAFLD/NASH, through the regulation of cell proliferation, inflammatory responses, and chemotaxis [38,39]. Several studies have shown that SARS-CoV-2 proteins might stimulate inflammatory responses through TLRs, such as TLR2 and TLR4-mediated signalling pathways [13,40]. These results further consolidate our identified HIRGs, suggesting their roles in the pathogenesis of CVD and COVID-19, as well as that they might be the therapeutic targets.

For patients with COVID-19, some therapeutic monoclonal antibodies (evusheld, as well as BRII-196 plus BRII-198) and antiviral drugs (paxlovid and molnupiravir) are widely used for improving outcomes [41,42,43]. However, there are certain concerns about the adverse effects, such as mutagenicity, birth defects, and rebound [44,45]. Besides, the effects of genetic variation of SARS-CoV-2 are a major concern for the control of the pandemic. Therefore, a comprehensive review of shared pathogenesis between CVD and COVID-19 may provide new insights into the future therapy development. In this study, we predicted the therapeutic SMCs, including solanine, desoxypeganine, alpha-linolenic-acid, CAY-10577, homochlorcyclizine, altretamine, NU-1025, TG100-115, and raltegravir based on the 12 hub genes, and further studies involving animals and clinical interventions will be required.

The immune system defends against pathogen invasion, but it can also drive life-threatening inflammatory responses. The seven HIRGs in this study were mainly positively associated with macrophages and neutrophils in the two diseases, suggesting their crucial roles in the regulation of the immune microenvironment. Patients with COVID-19 suffer a detrimental hyperinflammatory condition, with monocytes and macrophages as the main contributors [46]. Histopathological studies have also evidenced activated macrophages and neutrophils in numerous organs, such as the lungs, heart, and intestine of COVID-19 patients [47]. The infiltration of neutrophils and the formation of neutrophil extracellular traps (NETs) might exaggerate inflammatory responses and lead to the development of cardiovascular diseases through NET-mediated microthrombus formations and microvascular dysfunction [31,48]. Our findings demonstrate that these seven HIRGs could be candidates targeting inflammatory responses for novel therapeutic strategies. However, the current study also has some limitations. The bias from computational biology methods and samples from the database is limited, and further studies will incorporate more samples with both diseases to validate the seven identified HIRGs. In addition, in vivo studies are required to confirm the function of the identified transcriptional signatures.

## Figures and Tables

**Table 1:** The top 10 hub genes analyzed by cytoHubba.

MNC	EPC	DMNC	Degree	Closeness
TLR4	TLR4	ARG1	TLR4	TLR4
TLR2	TLR2	AIF1	TLR2	TLR2
MMP9	MMP9	THBD	MMP9	MMP9
CD163	CD163	CSF1R	CSF1R	CSF1R
CSF1R	CSF1R	CD163	CD163	CD163
CYBB	AIF1	CYBB	CYBB	CYBB
VWF	CYBB	VWF	VWF	VWF
AIF1	VWF	FPR2	AIF1	AIF1
ARG1	ARG1	BCL2A1	ARG1	ARG1
THBD	THBD	IL1R2	THBD	THBD
C5AR1	C5AR1	C5AR1	C5AR1	C5AR1
FPR2	FPR2	MMP9	MME	FPR2
BCL2A1	IL1R2	TLR2	FPR2	IL1R2
HBEGF	HBEGF	TLR4	HBEGF	BCL2A1
IL1R2	BCL2A1	HBEGF	NAMPT	BCL6

**Table 2:** Functional roles of immune-related hub genes.

Gene Symbol	Protein	CD Antigen Name	Function
C5AR1	C5a anaphylatoxin chemotactic receptor 1	CD88	Receptor for the chemotactic and inflammatory peptide anaphylatoxin C5a
MMP9	Matrix metalloproteinase-9		Matrix metalloproteinase that plays an essential role in local proteolysis of the extracellular matrix and in leukocyte migration
CYBB	Cytochrome b-245 heavy chain		Critical component of the membrane-bound oxidase of phagocytes that generates superoxide.
FPR2	N-fo-myl peptide receptor 2		Low affinity receptor for N-formyl-methionyl peptides, which are powerful neutrophil chemotactic factors
CSF1R	Macrophage colony-stimulating factor 1 receptor	CD115	Tyrosine-protein kinase that acts as a cell-surface receptor for CSF1 and IL34 and plays an essential role in the regulation of survival, proliferation, and differentiation of hematopoietic precursor cells, especially mononuclear phagocytes, such as macrophages and monocytes.
TLR2	Toll-like receptor 2	CD282	Cooperates with LY96 to mediate the innate immune response to bacterial lipoproteins and other microbial cell wall components.
TLR4	Toll-like receptor 4	CD284	Cooperates with LY96 and CD14 to mediate the innate immune response to bacterial lipopolysaccharide (LPS)

**Table 3:** Small molecules predicted with the common shared hub genes.

Rank	Score	Name	Description
1	−99.93	Solanine	Acetylcholinesterase inhibitor
2	−99.89	Desoxypeganine	Acetylcholinesterase inhibitor
3	−99.86	Alpha-linolenic-acid	Omega 3 fatty acid stimulant
4	−99.82	CAY-10577	Casein kinase inhibitor
5	−99.79	Homochlorcyclizine	Antihistamine
6	−99.75	Altretamine	DNA synthesis inhibitor
7	−99.71	NU-1025	PARP inhibitor
8	−99.65	TG100-115	PI3Kγ/PI3Kδ inhibitor
9	−99.62	Raltegravir	HIV integrase inhibitor

## Data Availability

Not applicable.
